# The Chemokine Receptor CCR7 Uses Distinct Signaling Modules With Biased Functionality to Regulate Dendritic Cells

**DOI:** 10.3389/fimmu.2020.00528

**Published:** 2020-04-15

**Authors:** José Luis Rodríguez-Fernández, Olga Criado-García

**Affiliations:** Centro de Investigaciones Biológicas Margarita Salas, Consejo Superior de Investigaciones Científicas, Madrid, Spain

**Keywords:** C–C chemokine receptor 7, signaling, leukocyte, MAPK pathway, RhoA pathway, PI3 K/Akt pathway, Chemotaxis (MeSH ID D002633)

## Abstract

Chemotaxis is a molecular mechanism that confers leukocytes the ability to detect gradients of chemoattractants. Chemokine receptors are well-known regulators of chemotaxis in leukocytes; however, they can regulate several other activities in these cells. This information has been often neglected, probably due to the paramount role of chemotaxis in the immune system and in biology. Therefore, the experimental data available on the mechanisms used by chemokine receptors to regulate other functions of leukocytes is sparse. The results obtained in the study of the chemokine receptor CCR7 in dendritic cells (DCs) provide interesting information on this issue. CCR7 guides the DCs from the peripheral tissues to the lymph nodes, where these cells control T cell activation. CCR7 can regulate DC chemotaxis, survival, migratory speed, cytoarchitecture, and endocytosis. Biochemical and functional analyses show: first, that CCR7 uses in DCs the PI3K/Akt pathway to control survival, the MAPK pathway to control chemotaxis, and the RhoA pathways to regulate actin dynamics, which in turn controls migratory speed, cytoarchitecture, and endocytosis; second, that these three signaling pathways behave as modules with a high degree of independence; and third, that although each one of these routes can regulate several functions in different settings, CCR7 promotes in DCs a functional bias in each pathway. The data uncover an interesting mechanism used by CCR7 to regulate the DCs, entailing multifunctional signaling pathways organized in modules with biased functionality. A similar mechanism could be used by other chemoattractant receptors to regulate the functions of leukocytes.

*“Divide and rule”* Attributed to Philip II of Macedon

## Introduction

Chemokine receptors regulate chemotaxis, a process that allows cells to detect gradients of chemoattractants. Based on this property, chemokine receptors, together with their ligands, serve as “address codes” that guide leukocytes to specific sites in the organism (1–3). Although chemoattraction is an important activity controlled by these receptors, they can regulate additional functions of leukocytes (4–6). This fact has been largely overlooked, probably due to the capital importance of chemoattraction in biology; consequently, the information on other functions of chemokine receptors is sparse (6). It is expected that non-chemotactic functions regulated by chemokine receptors may contribute to the efficient functioning of leukocytes in the immune system. Therefore, getting insight into the molecular mechanisms used to regulate these functions may allow the identification of novel targets to modulate the immune response.

C–C chemokine receptor 7, like all chemokine receptors, is included in the G protein-coupled receptor superfamily (6). CCR7 (ligands CCL19 and CCL21) is one of the chemokine receptors on which more functional information is available (6–8). We have studied the signaling pathways controlling CCR7-mediated functions in dendritic cells (DCs). It was found that, to control specific cellular functions of DCs, this receptor uses well-known signaling pathways that organize as signaling modules with biased functionality and limited crosstalk among them (9–13). Herein we describe the signaling components of these modules and discuss how they may regulate the functions of DCs.

## CCR7-Controlled Non-Chemotactic Activities May Contribute to the Efficiency of Dendritic Cells in the Immune System

Dendritic cells are leukocytes that are found in peripheral tissues in a differentiation state called immature, during which they display a high ability to detect, capture, and process antigens (14). After exposure to danger signals, including pathogens, toxic agents, or inflammatory cytokines, immature DCs undergo a complex differentiation program that transforms them into mature DCs (maDCs), which migrate to the lymph nodes (LNs), where they present antigens captured in the immature stage to antigen-specific T cells. As part of their differentiation program, the maDCs upregulate the expression of the chemokine receptor CCR7 that guides the maDCs to the LNs, attracted by CCL21 which is expressed in the afferent lymphatics vessels and by CCL19 and CCL21 which are expressed in stromal cells in the LNs (15, 16). CCR7 is crucial to guide the maDCs to the LNs, implying that its correct expression and function is important for adequate adaptive immune response (15, 17–21). Apart from chemoattraction (8, 22, 23), CCR7 regulates cytoarchitecture (13, 24), endocytosis (13, 25), survival (12), migratory speed (11, 26), adhesion (27), and differentiation in maDCs (28). Predictably, these non-chemotactic activities regulated by CCR7 contribute to the correct functionality of the maDCs in the immune system (6). It is expected that the enhanced survival, migratory speed, and differentiation may increase the number of antigen-loaded maDCs that reach the LNs. The increment in endocytosis may confer the maDCs migrating through the afferent lymphatic vessels, or located in the LNs, the ability to endocyte antigens, e.g., viral particles that can be subsequently presented to T cells (29, 30). Enhanced adhesion facilitates the migration of the maDCs through the afferent lymphatic vessels (27, 31). The induced changes in actin cytoarchitecture can regulate the motility of the maDCs that migrate toward the LNs and confer these cells their dendritic morphology (11, 13, 24). Pseudopod extensions increase the surface-area-to-volume ratio of the maDCs when compared with a spherical cell of equal volume (32), increasing the possibilities of contact with T cells. In summary, the different functions controlled by CCR7 may predictably contribute to a more effective maDCs in the immune system and to a better adaptive immune response (6). An important issue is the identification of the mechanisms used by CCR7 to regulate the cellular activities of the maDCs. In the following sections, we discuss recent experimental data that provide information on the molecules and the signaling mechanism involved in this process.

## CCR7-Dependent Survival Is Governed by a PI3K/AKT-Controlled Signaling Module

**FIGURE 1:**
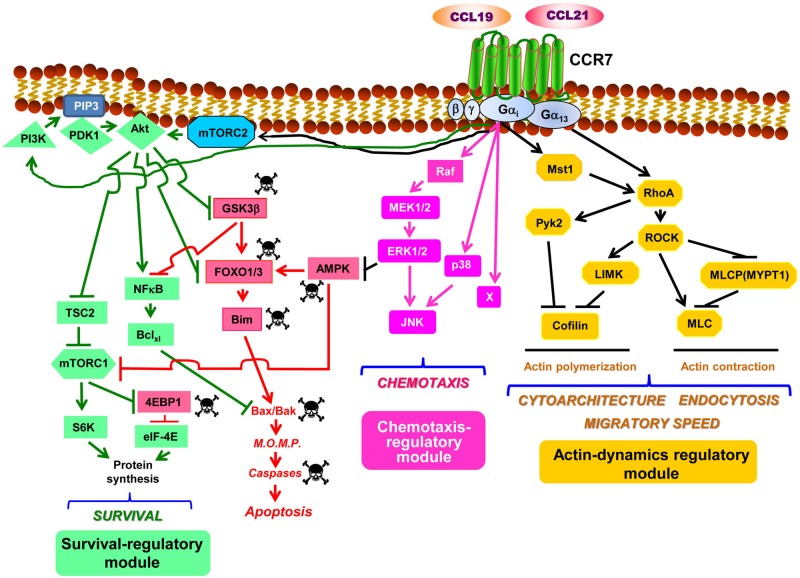
CCR7 uses signaling modules to regulate the functions of dendritic cells. Considering the sparse data within this field, the figure should be considered as a tentative model to be completed as additional experimental information becomes available. The model is largely based on the data presented in Table 1. Downstream of CCR7, the PI3K/Akt, the MAPKs, and the RhoA pathways organize as signaling modules that regulate survival, chemotaxis, and actin dynamics, respectively (see text for details). Abbreviations used: Akt, also known as Protein kinase B (PKB); AMPK, AMP-activated protein kinase; Bcl_*xl*_, B-cell lymphoma extra-large; Bim, Bcl-2-interacting mediator of cell death; 4EBP1, eukaryotic translation initiation factor 4E (eIF4E)-binding protein 1; MEK1/2, MAPK/ERK kinase 1 and 2; ERK1/2, extracellular signal-regulated kinase 1 and 2; mTORC1, mechanistic target of rapamycin (mTOR) complex 1; FOXO1/3, forkhead box protein O1 and O3; GSK3β, glycogen synthase kinase 3β; JNK, c-Jun N-terminal kinase; LIMK, LIM domain kinase; mTORC2, mTOR complex 2; MLC, myosin light chain; M.O.M.P., mitochondrial outer membrane permeabilization; Mst1, mammalian sterile 20-like kinase 1; MYPT1, myosin phosphatase target subunit 1; NFκB, nuclear factor-κB; PDK1, phosphoinositide-dependent kinase-1; PIP3, phosphatidylinositol (3,4,5)-trisphosphate; PI3K, phosphatidylinositol 3-kinase; Pyk2, proline-rich tyrosine kinase 2; RhoA, Ras homolog family member A; ROCK, Rho-associated protein kinase; S6, ribosomal protein S6; S6K, S6 kinase; TSC2, tuberous sclerosis complex 2. The skull and crossbones symbol indicates molecules that promote apoptosis.

When maDCs are kept in serum-free medium, they initiate an apoptotic program that leads to their demise (12). This simple experimental setting is useful to identify the receptors that inhibit cellular apoptosis and the intracellular pathways involved (12, 33). The stimulation of maDC kept in serum-free medium with any of the ligands of CCR7, CCL19, or CCL21 slows down the apoptosis of these cells, indicating that this receptor induces anti-apoptotic intracellular signaling (12). Using this experimental strategy, it was also found that the kinases AMPK and GSK3β played pro-apoptotic roles *in vitro* and *in vivo* because a forced increase or decrease of their activities enhanced or impaired apoptosis in maDCs (9, 10, 34) (Figure 1). Moreover, it was found that AMPK promotes apoptosis in maDCs by inhibiting the mechanistic target of rapamycin complex 1 (mTORC1), a kinase complex that promotes survival in maDCs (see below) (10). Both AMPK and GSK3β induce the activation of the transcription factors FOXO1/3, which controls the pro-apoptotic Bcl2 family member Bim (9, 10, 12, 35). Moreover, active GSK3β also prevents the activation of anti-apoptotic transcription factor NFκB, which controls the transcription of the anti-apoptotic Bcl2 family member Bcl_*xl*_ (9) (Figure 1). The balance between pro-apoptotic and anti-apoptotic Bcl2 family members determines whether a cell becomes apoptotic or survives (36). An excessive increase in pro-apoptotic (e.g., Bim) over pro-survival (e.g., Bcl_*xl*_) Bcl2 members induces the activation of the mitochondria gatekeepers Bax/Bak (36, 37), resulting in mitochondrial outer membrane permeabilization and liberation of cytochrome c from the intermembrane space of the mitochondria, which leads to caspase activation and apoptosis (Figure 1) (36).

**TABLE 1:** Experimental data support a high degree of independence between the different CCR7-regulated modules controlling the functions of the dendritic cells.

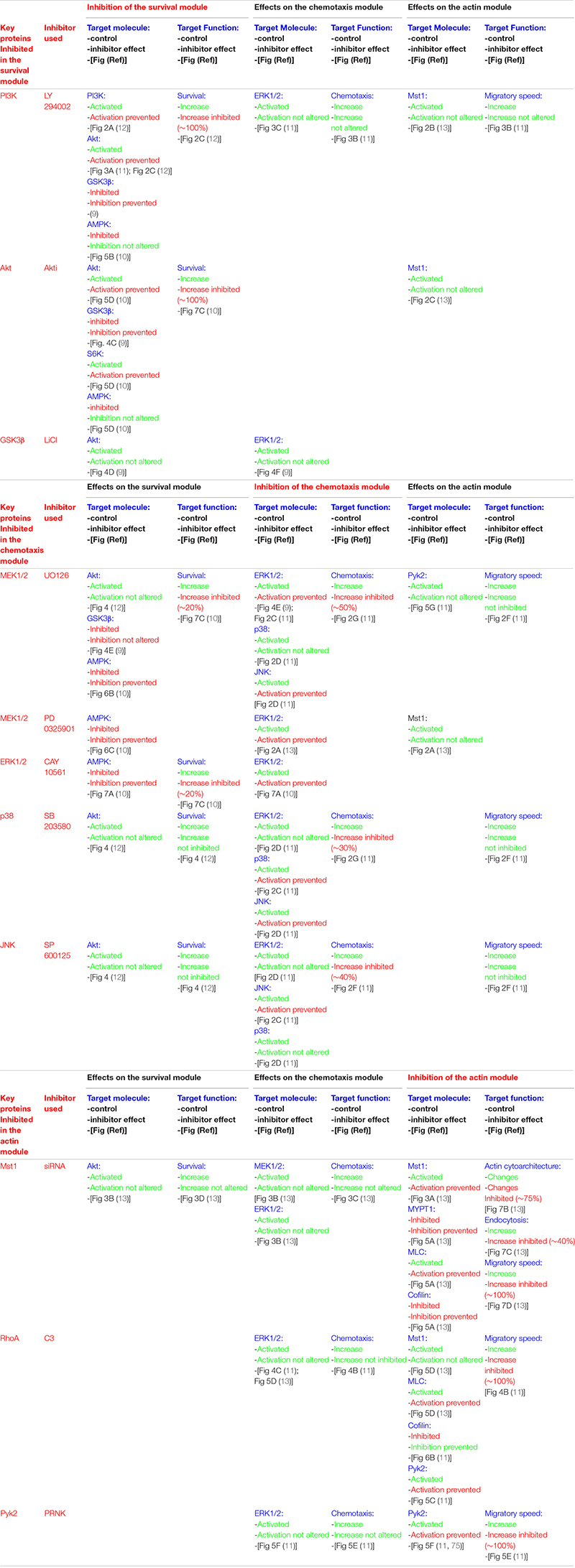

When CCR7 was stimulated in the maDCs that were in serum-free medium, it was observed that the pro-apoptotic signaling described above was turned off because this receptor induced the activation of the signaling axis PI3K/Akt (9, 10, 12, 19, 38, 39) which, as shown below, is a core component of a pro-survival pathway in these cells (Figure 1). The Gi family of G proteins and, particularly, the Gβγ complex (12), a dimer associated to the Gα subunit to form a heterotrimeric G protein, mediate the CCR7-dependent activation of the PI3K/Akt pathway (10, 12). This pathway induces survival in multiple cell types due to the ability of its signaling components, particularly Akt, to switch off pro-apoptotic molecules and turn on pro-survival signals (40, 41). Upon the CCR7-dependent activation of Akt in maDCs, this kinase directly phosphorylates and inhibits the transcription factor FOXO1/3 which, as mentioned above, controls the expression of pro-apoptotic Bim (9). Active Akt further prevents apoptosis by phosphorylating/inhibiting GSK3β which, as mentioned before, promotes the activation of pro-apoptotic FOXO1/3 and also the inhibition of pro-survival NFκB (9) (Figure 1). Akt also induces the activation of the transcription factor NFκB, which regulates Bcl_*xl*_ (9, 12) that, as indicated above, protects the cells from apoptosis by opposing pro-apoptotic Bim (Figure 1). Akt can also enhance cell survival by inducing the activation of mTORC1, which stimulates translation, a process that promotes survival in maDCs (42) (Figure 1). Active mTORC1 stimulates translation by inducing phosphorylation/inactivation of the eukaryotic translation initiation factor 4E (eIF4E)-binding protein 1 (4E-BP1), which retains eIF-4E inhibited. After the phosphorylation of 4E-BP1, eIF-4E is released and becomes part of the translation initiation complex (10, 42). Moreover, mTORC1 also activates translation by phosphorylating/activating ribosomal S6 kinase (S6K), which subsequently phosphorylates the protein targets involved in translation, including the ribosomal protein S6 (Figure 1). The combined effects of the activation of Akt, which leads to the up-regulation of Bcl_*xl*_, the inhibition of Bim and, in addition, the activation of mTORC1, which promotes an increase in protein synthesis, contribute to extend the survival of the maDCs (10, 42). The prior results indicate that the PI3K/Akt pathway mediates CCR7-dependent survival. Regarding the contribution of other pathways to the regulation of survival, a modest contribution of the chemotaxis pathway was observed because the inhibition of MEK1/2/ERK1/2, two key regulators of chemotaxis (see below), reduced by ∼20% the pro-survival effects induced by CCR7 (10). MEK1/2/ERK1/2 may exert this moderate pro-survival effect by inhibiting the pro-apoptotic kinase AMPK (10) (Table 1 and Figure 1).

The inhibition of the PI3K/Akt pathway strikingly failed to affect the CCR7-dependent MAPK pathway (Table 1), the RhoA pathway, or the functions regulated by these two routes in maDCs (Table 1) (9, 10, 12). A lack of effect of PI3K/Akt on CCR7-regulated chemotaxis (19, 39) or endocytosis (see below) has also been reported by other groups (39). These results suggest that the PI3K/Akt pathway constitute a signaling module that largely controls CCR7-induced survival, but no other CCR7-mediated functions in maDCs (see below). This is interesting because the PI3K/Akt pathway also regulates chemotaxis, proliferation, and metabolism in other cells (43, 44). This functional bias is also observed in the other two pathways controlled by CCR7 in maDCs (see below). In summary, the data indicate that CCR7 controls in maDCs a PI3K/Akt-controlled signaling module that regulates survival, but not chemotaxis or actin dynamics (see below).

## CCR7-Dependent Chemoattraction Is Governed by a MAPK- Controlled Signaling Module

Experimental evidence indicates that CCR7-dependent chemotaxis is largely regulated by a signaling module formed by Raf and the MAPKs family members MEK1/2, ERK1/2, p38, and JNK in maDCs (11, 39). As shown for CCR7-induced survival, the activation of both the MAPK pathway and chemotaxis is regulated in maDCs by Gi proteins and Gβγ dimers (11). Previously, Gβγ dimers have been shown to regulate chemotaxis in other chemokine receptors and cells (45). Although it has not been experimentally analyzed if CCR7 induces the activation of Ras in maDCs, it is possible that this GTPase may mediate the effects of CCR7 on the MAPK pathway because Ras is an upstream regulator of the Raf-MEK1/2-ERK1/2 pathway (46), and different chemokine receptors can induce the activation of this GTPase (47–49). Moreover, upon G-protein coupled receptor activation, Gi and Gβγ dimers mediate, like in maDCs, the Ras-dependent activation of MAP kinase pathway (50, 51). In summary, the results indicate that the MAPK pathway controls CCR7-dependent chemotaxis in maDCs (11). However, the complete inhibition of ERK1/2, p38, and JNK does not abrogate chemotaxis, suggesting that additional regulator/s, denoted as “X” in Figure 1, may also contribute to the regulation of this pathway (11). The inhibition of key molecular components of the MAPK pathway does not affect dramatically the CCR7-dependent PI3K/Akt pathway and survival or the RhoA pathway and the function associated to it (actin-regulated functions, including cytoarchitecture, endocytosis, and migratory speed; see below) (Table 1). Other authors have also shown that the stimulation of CCR7 induces the activation of JNK and that the inhibition of this MAPK blocks chemotaxis, but not the endocytosis in maDCs (39), supporting the independence of the RhoA pathway (see below) of the chemotaxis regulatory module. In summary, the results suggest that the MAPK pathway may constitute a signaling module that displays a high degree of independence since it seems independent of the module that regulates CCR7-regulated actin dynamics and displays only a very modest contribution to the regulation of CCR7-controlled survival (Figure 1). Another interesting aspect that emerges from these results is the high degree of functional bias of the MAPK pathway, which seems to regulate mainly CCR7-controlled chemotaxis and only modestly survival in maDC, although it is a potent regulator of survival and proliferation in other contexts (52).

The data suggesting that the MAPK pathway mediates CCR7-dependent chemotaxis in maDCs is consistent with prior data showing that Ras, an upstream regulator of the MAPKs (46), is a regulator of chemotaxis in response to N-formyl-L-methionyl-L-leucyl-L-phenylalanine (fMLP) in neutrophils (53, 54) and to cyclic-adenosine monophosphate (cAMP) in *Dictyostelium discoideum* (55, 56). Moreover, in *Dictyostelium*, Ras activation takes place independently of PI3K (55), which reminds of the observed independence between the CCR7-regulated activation of MAPK and the PI3K/Akt pathways described in maDCs. In summary, the results indicate that CCR7 controls in maDCs a MAPK-regulated signaling module, which selectively controls chemotaxis, independently of the survival and actin-regulatory modules (see below).

## CCR7-Dependent Changes in Actin Dynamics Is Governed by a RhoA-Controlled Signaling Module

Our studies indicate that the CCR7-induced stimulation of migratory speed, endocytosis, and changes in cytoarchitecture in maDCs is mediated by the RhoA pathway (11, 13). Thus RhoA, a key regulator of actin organization in multiple cells, including maDC (57–64), would govern the actin dynamic changes involved in the control of the aforementioned activities. It has been suggested that RhoA does not mediate CCR7-induced morphological changes and endocytosis in murine maDCs, which would be controlled instead by Cdc42 and Rac (24, 25). These discrepancies could be due to species differences [murine maDCs (24, 25) vs. human maDCs (11, 13)] or caused by the maturation stimulus used for the DCs [LPS (24, 25) vs. TNFα (11, 13)]. This issue will have to be settled in future studies. Unlike CCR7-dependent survival and chemotaxis, which depend largely on Gi proteins, CCR7-dependent changes in actin dynamics were found to be regulated both by Gi and G_13_ family of G proteins (Figure 1). Interestingly, the kinase Mst1 connects Gi with RhoA, which is also downstream of G_13_ (13) (Figure 1). These data are consistent with prior results indicating that the G_12__/__13_ proteins control RhoA (65). RhoA effects are mediated by a pathway that controls actin dynamics, including actin polymerization (ROCK-LIMK-cofilin) and contraction (ROCK/MLCP/MLC) (57, 59, 61) (Figure 1). It was also observed that, downstream of CCR7, RhoA controls the activation of the tyrosine kinase Pyk2 (11) (Figure 1), suggesting that this kinase can also mediate the effects of RhoA on the actin cytoskeleton. Accordingly, other authors have suggested that Pyk2 is activated downstream of G_13_ and that it is involved in the control of leukocyte motility and cytoarchitecture (66, 67). The selective blocking of the molecular components of the RhoA pathway in mDCs results in the inhibition of CCR7-dependent migratory speed, endocytosis, and alterations of the cytoarchitecture (11, 13), suggesting that RhoA-controlled actin mediates these functions (58, 61). In summary, CCR7 controls two axes, namely, CCR7/G_13_ and CCR7/G_*i*_/Mst1, that converge on RhoA, which is upstream of a pathway that controls the actin dynamics involved in the regulation of migratory speed, endocytosis, and cytoarchitecture (Figure 1) (11, 13, 57, 60). As shown for the other modules, the inhibition of specific signaling components of this pathway failed to affect the chemotaxis or survival of the signaling components controlling these functions, supporting the independence of the CCR7-dependent RhoA-regulated signaling module (Table 1) (11, 13). Supporting that the actin dynamics regulatory module is independent of the chemotaxis module, it has been shown that the inhibition of the kinase ROCK fails to block the activation of the chemotaxis regulator JNK in maDCs (39). The results together suggest that the CCR7-regulated RhoA pathway behaves as a signaling module that displays a high degree of independence in maDCs. As shown with the other two modules, although in addition to actin dynamics, the RhoA pathway may regulate other cell functions, including survival and proliferation (62); however, CCR7 in maDCs apparently regulates largely actin dynamics. In summary, the RhoA-regulated module controls selectively CCR7-dependent actin dynamics and the cellular activities associated to it, including migratory speed, endocytosis, and cytoarchitecture.

Finally, the described independence between the chemotaxis and actin dynamics regulatory modules suggests that chemotaxis and motility are different functions. The following results further support this concept. Using microfluidic devices, it has been shown that perturbing actin dynamics with actin and myosin inhibitors in mouse maDCs affects the migratory speed, but not the chemotaxis in response to CCL19 (68). The actin-associated protein mDia, which regulates actin dynamics, mediates migratory speed, but it is dispensable for 3D chemotaxis in response to CCL21 in murine maDCs (69, 70). In response to the external gradients of cAMP, in *D. discoideum*, a polarized localization of Ras is observed, and in neutrophils exposed to the gradients of fMLP, a polarization of PI phosphatidylinositol (3,4,5)-trisphosphate (PIP3) also takes place. However, in *Dictyostelium* and human neutrophils exposed to these chemoattractants, Ras and PIP_3_ still polarize, even when the cells were immobilized either on highly adherent substrates or by disrupting their actin cytoskeleton with latrunculin (53, 55). It has been shown that, although the actin-associated leading edge protein Arp2/3, which regulates actin dynamics, is critical for lamellipodial formation and cell motility in fibroblast and cancer cell lines, it is, however, dispensable for chemotaxis (71, 72). These examples suggest that perhaps it is more appropriate to define chemotaxis as “chemoattractant sensing” to separate it from motility, which could be a different cell activity.

## Concluding Remarks

Herein we discuss experimental findings indicating that CCR7 activates three signaling pathways in maDCs, namely, the PI3K/Akt, the MAPK, and the RhoA pathways, which largely regulate survival (12), chemotaxis (11), and actin dynamics (11, 13), respectively. The results obtained suggest a high degree of independence between these pathways, although it is not complete because at least the chemotaxis and the survival modules are connected, with the former controlling modestly the latter. Albeit each one of the three pathways can regulate several functions in different contexts (43, 44, 52, 62), CCR7 seems to select only one activity in maDCs. The molecular mechanisms supporting the independence and biased functionality of these pathways are not known. CCR7 regulates in maDCs other signaling molecules not analyzed in this review, e.g., cyclic AMP, calcium, phospholipase C, Src, and others (11, 19, 73). Future studies will determine their roles in the modules described or in others described in the future. Finally, the independent modular organization described could be one among several strategies used by chemokine receptors to regulate leukocyte functions because, for instance, the receptor CXCR4 uses redundant signaling to control survival and chemotaxis in maDCs (33). In summary, the information gathered point out an interesting mechanism that could be used by multifunctional chemokine receptors to regulate the functions of leukocytes.

## Author Contributions

JR-F conceived and wrote the manuscript. OC-G performed important contributions to the manuscript and the figure and designed the table as presented.

## Conflict of Interest

The authors declare that the research was conducted in the absence of any commercial or financial relationships that could be construed as a potential conflict of interest.
